# Genome-wide mapping of imprinted differentially methylated regions by DNA methylation profiling of human placentas from triploidies

**DOI:** 10.1186/1756-8935-4-10

**Published:** 2011-07-13

**Authors:** Ryan KC Yuen, Ruby Jiang, Maria S Peñaherrera, Deborah E McFadden, Wendy P Robinson

**Affiliations:** 1 Department of Medical Genetics, University of British Columbia, 2329 West Mall, Vancouver, BC, V6T 1Z4, Canada; 2 Child & Family Research Institute, 950 West 28th Ave, Vancouver, BC, V5Z 4H4, Canada; 3 Department of Pathology, University of British Columbia, 2329 West Mall, Vancouver, BC, V6T 1Z4, Canada

## Abstract

**Background:**

Genomic imprinting is an important epigenetic process involved in regulating placental and foetal growth. Imprinted genes are typically associated with differentially methylated regions (DMRs) whereby one of the two alleles is DNA methylated depending on the parent of origin. Identifying imprinted DMRs in humans is complicated by species- and tissue-specific differences in imprinting status and the presence of multiple regulatory regions associated with a particular gene, only some of which may be imprinted. In this study, we have taken advantage of the unbalanced parental genomic constitutions in triploidies to further characterize human DMRs associated with known imprinted genes and identify novel imprinted DMRs.

**Results:**

By comparing the promoter methylation status of over 14,000 genes in human placentas from ten diandries (extra paternal haploid set) and ten digynies (extra maternal haploid set) and using 6 complete hydatidiform moles (paternal origin) and ten chromosomally normal placentas for comparison, we identified 62 genes with apparently imprinted DMRs (false discovery rate <0.1%). Of these 62 genes, 11 have been reported previously as DMRs that act as imprinting control regions, and the observed parental methylation patterns were concordant with those previously reported. We demonstrated that novel imprinted genes, such as *FAM50B*, as well as novel imprinted DMRs associated with known imprinted genes (for example, *CDKN1C *and *RASGRF1*) can be identified by using this approach. Furthermore, we have demonstrated how comparison of DNA methylation for known imprinted genes (for example, *GNAS *and *CDKN1C*) between placentas of different gestations and other somatic tissues (brain, kidney, muscle and blood) provides a detailed analysis of specific CpG sites associated with tissue-specific imprinting and gestational age-specific methylation.

**Conclusions:**

DNA methylation profiling of triploidies in different tissues and developmental ages can be a powerful and effective way to map and characterize imprinted regions in the genome.

## Background

Genomic imprinting is a phenomenon in which one of the two alleles of a gene is expressed in a parent-of-origin manner [1]. Imprinted genes are thought to be particularly important to placental and foetal growth and development and may help regulate growth in response to maternal and foetal signals *in utero *[2]. To date, around 60 imprinted genes have been identified in humans (http://www.geneimprint.com), largely after first being identified in mice or through characterization of specific imprinting disorders such as Prader-Willi syndrome and Angelman syndrome or Beckwith-Wiedemann syndrome. However, many genes are imprinted in mice but are not known to be in humans, for example, *Impact *[3]. Furthermore, many genes are imprinted only in specific tissues, for example, *Ube3a*, which is maternally expressed in the brain but biparentally expressed in other tissues [4], or may be polymorphically imprinted, for example, *IGF2R *[5]. These issues complicate the discovery and characterization of imprinted genes in humans.

The importance of imprinted genes for placental and foetal development was initially demonstrated in mice by observations that parthenogenetic embryos (maternal origin, digynic diploid) could show embryonic differentiation but failed to form extraembryonic components [6]. In contrast, androgenetic embryos (paternal origin, diandric diploid) had poorly developed embryos, but the trophoblasts showed extensive proliferation [7]. The parallel observations in humans are ovarian teratomas (parthenogenetic), which are a rare form of tumour that consists of a variety of embryonic tissues or organs but no placental tissues, and complete hydatidiform moles (CHMs) (androgenetic), which consist of abnormal placental growth characterized by trophoblast hyperplasia but no (or rare) embryonic structures. The parental conflict theory developed to explain the evolution of imprinted genes [8] suggests that paternally expressed genes tend to promote growth of the offspring at the expense of the mother, while maternally expressed genes act as growth-limiting factors to conserve maternal resources [8].

Most imprinted genes possess differentially methylated regions (DMRs) whereby allelic methylation depends on the parent of origin [1]. DMRs established through the germline are called 'gametic' or 'primary' DMRs. These often coincide with imprinting control regions (ICRs), which regulate gene expression and further epigenetic modifications [9-11]. Their methylation status is thought to be maintained in all somatic lineages once acquired. Other DMRs, called 'somatic' or 'secondary' DMRs, are established after fertilization and may be tissue-specific [10,11].

Since most imprinted genes contain DMRs, comparing DNA methylation profiles between tissues with unbalanced parental constitutions provides an approach to identify and characterize imprinted genes in the genome. One approach is to compare the DNA methylation profile of paternally derived CHMs to that of maternally derived ovarian teratomas [12]. Indeed, several novel imprinted genes have been identified previously by using this strategy [13,14]. However, such comparisons are limited by the fact that the tissues present in ovarian teratomas and CHMs are highly abnormal and are not of comparable origin, with teratomas being embryonic and CHMs being strictly placental. Many differences may reflect tissue-specific methylated genes, since tissue-specific DMRs are numerous and are established in early pregnancy [15]. CHMs also present with highly proliferative trophoblasts that can lead to increased risk of choriocarcinoma, and hypermethylation of nonimprinted genes has been reported in CHMs [16].

In humans, triploidy (the presence of three complete haploid genomes) occurs spontaneously in 2% to 3% of pregnancies, and, while such pregnancies frequently end in miscarriage, they can survive into the foetal period and, very rarely, to term [17]. We propose that a comparison between diandric and digynic triploidies, in which development is much less severely altered than in CHMs and teratomas, provides a powerful approach for the identification and characterization of imprinted genes in the human genome. The diandric triploid phenotype (two paternal plus one maternal haploid genomes) is characterized by a normal-sized or only moderately growth-restricted foetus with a large and cystic placenta with trophoblast hyperplasia, while the digynic triploid phenotype (two maternal plus one paternal haploid genomes) is characterized by an intrauterine growth-restricted foetus and a very small placenta with no trophoblast hyperplasia [17]. Importantly, embryo and foetal development are largely similar between diandric and digynic triploidy, with growth differences likely arising largely as a consequence of differences in placental function [18]. Furthermore, while small, digynic placentas have a grossly normal structure. Diandric placentas show features similar to a CHM, but their development is much less severely altered than in a CHM, and the placenta can support growth of a foetus at least to some degree.

Although it was previously suggested that DNA methylation may be less important in regulating imprinting in placental tissue as compared to foetal tissue, we recently demonstrated that the DNA methylation status of many known imprinted DMRs is strictly maintained in triploid placentas and can be used to distinguish diandric from digynic triploidy [19]. Therefore, in the present study, we compared the DNA methylation profiles of placentas from diandric and digynic triploidies using the Infinium HumanMethylation27 BeadChip array (Illumina, Inc., San Diego, CA, USA), which targets over 27,000 CpG loci within the proximal promoter regions of approximately 14,000 genes [20]. Methylation levels in chromosomally normal placentas, CHMs and maternal blood samples were used as reference points for comparison. Using this strategy, we identified the majority of known imprinted ICRs on the array and many novel imprinted DMRs in the genome. For a subset of genes, we identified expressed polymorphisms and informative mother-placenta pairs, which were used to demonstrate parent-of-origin biases in allelic expression. We also demonstrated that complex DNA methylation domains that regulate imprinted genes can be mapped by comparing the methylation patterns in different tissues and different gestational ages of placentas.

## Results

### DNA methylation profile analysis in placenta and blood samples

**Figure 1:**
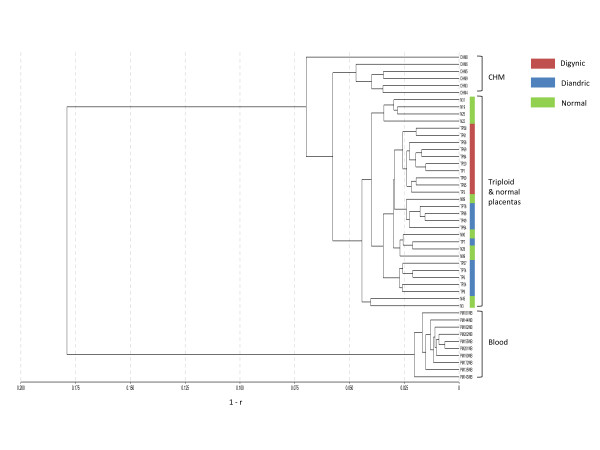
**Unsupervised clustering of triploid and normal placentas with CHMs and blood samples demonstrates that each tissue type has a distinct methylation profile**. Sample names are shown with labelling of corresponding tissue types. Samples were clustered by hierarchical clustering of β values based on 1-*r *(Illumina GenomeStudio software), where *r *represents the correlation coefficient between samples. Digynic triploids are indicated by red boxes, diandric triploids by blue boxes and normal placentas by green boxes.

To generate DNA methylation profiles from triploidies, we assayed placental DNA from ten diandric and ten digynic triploidies on the Illumina Infinium HumanMethylation27 BeadChip panel. In addition, ten chromosomally normal placentas, 6 CHMs (diandric diploid, no maternal contribution) and ten maternal whole-blood samples were included for comparison. After background adjustment and normalization, we performed unsupervised hierarchical clustering with all the samples based on a distance measure of 1-*r*, where *r *is the Pearson correlation coefficient between different samples. This revealed three distinct groups of clusters: CHMs, triploid and normal placentas, and blood (Figure 1). The blood cluster is more distant from the two other clusters of placentas, confirming that there are many DNA methylation differences between blood and placenta [21-23]. Although CHMs are trophoblast-derived, they show a distinct methylation profile from the triploid and normal placentas, which probably reflects not only the lack of a maternal genome but also the abnormal development of such tissue. Within triploid and normal placentas, digynic and diandric triploid placentas are clearly separated by their methylation profiles, but, interestingly, they are not separated from the chromosomally normal placentas (Figure 1). This suggests that methylation profiles of triploid placentas closely resemble those of chromosomally normal placentas, but that digynic and diandric triploid placentas have distinguishing DNA methylation differences.

Although clustering can be biased by gender differences resulting from inactivation of an X chromosome in females (that is, higher methylation of the X chromosome CpG islands in female than in male samples) [22,24], there is no preferential clustering of samples by gender within the triploid and normal placenta cluster (Figure S1A in Additional file 1). There is a small difference in gestational age (about three weeks apart on average) between diandric and digynic placentas (*P *< 0.01) (Table S1 in Additional file 2), but this also cannot explain the distinct clustering patterns, since the gestational ages of the two groups are largely overlapping (Table S1 in Additional file 2).

We further compared the average DNA methylation of probes between the five sample groups (digynic triploid placentas, diandric triploid placentas, normal placentas, CHMs and blood) (Figure S1B in Additional file 1). As expected, the correlation of average probe methylation values between different sample groups is consistent with that observed in the cluster analysis. In general, blood has the most distinct DNA methylation profile with a greater number of highly methylated probes (Figure S1B in Additional file 1). Triploid and normal placentas are highly correlated with regard to their methylation profiles (*r *= 0.99), while CHMs are more similar to diandric and normal placentas (*r *= 0.98) than to digynic placentas (*r *= 0.96).

### Comparison of DNA methylation profiles between placentas from diandric and digynic triploidies

After comparing methylation between diandric and digynic placentas by performing Student's *t*-test for all probes, nearly 2,500 probes were identified with a *P *value < 0.01, which is nearly ten times more than expected by chance (Figure S1C in Additional file 1). To adjust for multiple testing and identify candidates with a very high likelihood of representing true differences, we used a stringent cutoff of <0.1% false discovery rate (FDR) by using the Significance Analysis of Microarrays (SAM) program with 1,000 permutation comparisons for each sample [25]. To further focus on the most meaningful differences, we also considered only probes with more than 15% absolute magnitude difference between the mean methylation of diandric and digynic triploidies. While we expected a theoretical difference of 33.3% for imprinted sites, we used a lower cutoff because we have observed that the actual methylation difference may vary for some known imprinted genes [19] and that there may be biases in the Illumina array that result in a nonlinear relationship between the estimated methylation β value and actual methylation. In total, 122 probes were identified with <0.1% FDR and average absolute methylation difference >15% (average absolute Δ β >0.15 from the Illumina array). Probes with higher average methylation in diandric than digynic triploidies were designated putative paternal differentially methylated loci (DML), and probes with higher average methylation in digynic than diandric triploidies were designated putative maternal DML. Plotting DNA methylation of putative DML in all samples from diandric against digynic triploidies showed a clear separation of methylation values of paternal and maternal DML (Figure S1D in Additional file 1), suggesting that most of the identified differentially methylated probes are consistently methylated within each sample group without much overlap as expected on the basis of our application of stringent statistical criteria.

**Figure 2:**
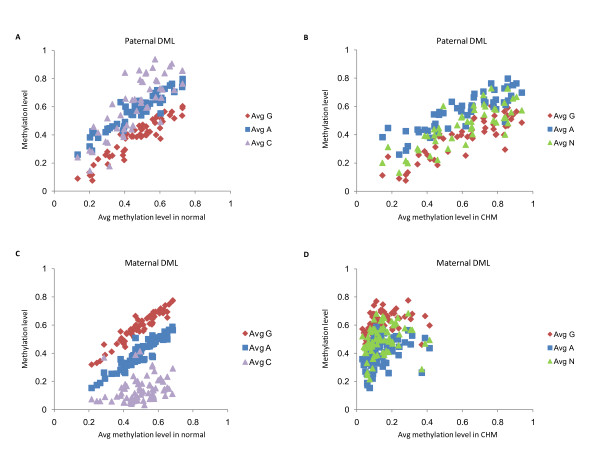
**Scatterplots of average methylation of paternal and maternal DML**. **(A) **and **(C) **Average methylation values in normal placentas (*x*-axis) plotted against digynic triploids (Avg G), diandric triploids (Avg A) and CHMs (Avg C) show high correlation. **(B) **and **(D) **Average methylation values in CHMs (*x*-axis) plotted against digynic triploids (Avg G), diandric triploids (Avg A) and normal placentas (Avg N).

As some methylation differences between diandric and digynic triploids could theoretically arise as a result of secondary effects, such as altered cell composition, the validity of the identified putative imprinted DML was further evaluated by verifying that the methylation levels of diandric CHMs and chromosomally normal placentas fit the expected pattern (Figure 2). The average methylation in CHMs was closer in value to that of diandric triploidies (Figures 2A and 2C), while that for normal placentas fell between that for diandric and digynic triploidies for the majority of putative DML (Figures 2B and 2D) as would be expected for imprinted DMRs. The putative maternal DML were more strongly correlated with normal placentas than paternal DML, while putative paternal DML tended to have higher correlation with CHMs than maternal DML (Figure 2 and Figure S1E in Additional file 1). CHMs showed particularly low correlation for maternal DML compared with other placental groups, which was largely due to the low average methylation of putative maternal DML in CHMs as well as more variability in values for CHMs (Figure 2D).

**Figure 3:**
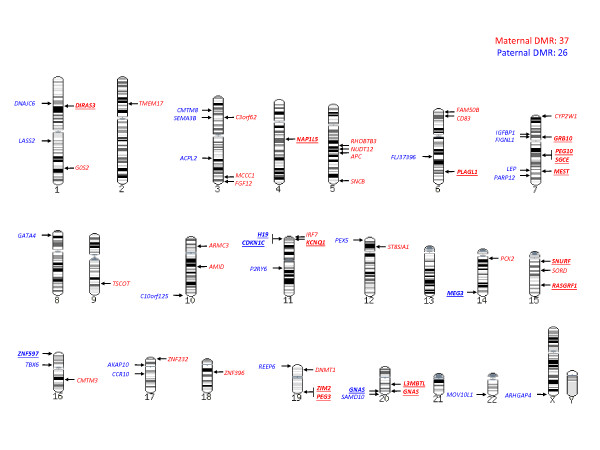
**Location of the 63 identified DMRs in the genome**. Relative location of the identified 37 maternal DMRs and 26 paternal DMRs are shown in the human genome according to the genomic sequence released in 2006 in the UCSC Genome Browser database (hg18). Paternal DMRs are highlighted in blue and maternal DMRs are highlighted in red. Known imprinted genes are boldfaced and underlined. Chromosome 7 contains the highest number of DMRs (nine DMRs), while there are no DMRs identified on chromosomes 13, 21 and Y.

Fourteen probes failed to follow the expected relative methylation patterns between the between groups (normal placentas with an average methylation level between that for diandric and digynic placentas and CMHs with an average methylation level closer to that in diandric placenta), and these loci were eliminated as candidates for further analysis. This yielded a final list of 108 identified putative DML that are associated with 63 different DMRs from 62 genes (one gene with both paternal and maternal DML) (Table S2 in Additional file 2). Of the 63 DMRs, 37 are maternally methylated and 26 are paternally methylated (Figure 3). These imprinted DMRs are distributed across the whole genome, with chromosome 7 containing the highest number (nine DMRs), while chromosomes 13, 21 and Y are the only chromosomes for which no DMRs were identified (Figure 3).

As copy number variation (CNV) can be a potential bias for methylation [26], we referred to the UCSC Genome Brower database (hg18) (http://www.genome.ucsc.edu) and found that the locations of 37 of the 108 probes overlap with known CNVs (Table S2 in Additional file 2). However, any effect of the CNVs on methylation of the candidate sites identified by our criteria was minimal, since the methylation of maternal and paternal DML were clearly separated from each other without much overlap (Figure S1D in Additional file 1). Similarly, differences between the two groups are unlikely to be caused by differences in genetic sequence polymorphisms that influence methylation, as this would require all ten diandric placentas, by chance, to be of a different genotype from all ten dygynic placentas.

### Validation of DNA methylation patterns of identified putative imprinted DMRs

The microarray included 374 CpG sites in the promoter regions of 59 genes that have previously been reported to be imprinted in humans based on the literature [12] and information in Internet databases (http://igc.otago.ac.nz/ and http://www.geneimprint.com/) (Table S3 in Additional file 2). For nine of these genes (*PRIM2A*, *IGF2R*, *TFP12*, *COPG2*, *KLF14*, *ABCA1*, *INPP5F*, *IGF2AS *and *BLCAP*), the included CpG sites were unmethylated (or very lowly methylated) in the normal human placentas, as well as in the triploid placentas (Table S3 in Additional file 2). The majority (39 of 50) of the remaining genes showed differences between diandric and digynic triploids at one or more of the associated CpG sites (*t*-test, *P *< 0.05), though typically not all CpG sites were differentially methylated.

Among the 62 genes identified with parent-of-origin-dependent DMRs (using the stricter criteria of <0.1% FDR and absolute average mean difference >15%), 18 are known imprinted genes associated with 15 distinct DMRs. Two of the identified DMRs, associated with the imprinted genes *CDKN1C *and *RASGRF1*, have been reported only in mice and not in humans [3,12] (Table 1). While our strict selection criteria yielded only 18 of the 39 known imprinted genes that were statistically significantly different between diandric and digynic triploidies using an uncorrected *P *< 0.05 (Table S3 in Additional file 2), the missed cases were largely due to the mean difference being less than the 15% average methylation difference cutoff. Eleven of the fifteen imprinted DMRs are known to be ICRs with a parental origin of methylation concordant with what we observed based on the comparison of triploidies (Table 1).

**Table 1:** Identified DMRs with known imprinted DMRs^a^

Location	Gene	Expressed allele	ICR	Known DMR	Identified DMR
1p31	*DIRAS3*	P	-	M	M
4q22.1	*NAP1L5*	P	M	M	M
6q24	*PLAGL1*	P	M	M	M
7p12	*GRB10*	M/P^b^	M	M	M
7q21.3	*PEG10*/*SGCE*	P	M	M	M
7q32.2	*MEST*	P	M	M	M
11p15	*CDKN1C*	M	-	P^c^	P
11p15	*H19*	M	P	P	P
11p15	*KCNQ1*^d^	M	M	M	M
14q32	*MEG3*	M	P	P	P
15q11-q12	*SNURF*	P	M	M	M
15q24	*RASGRF1*	P	-	P^c^	M
16p13	*ZNF597*	M	-	-	P
19q13.43	*PEG3*/*ZIM2*	P	M	M	M
20q13	*GNAS *(NESP)	M	-	P	P
20q13	*GNAS *(XL)	P	M	M	M
20q13	*L3MBTL*	P	-	M	M

^a^DMR, differentially methylated region; ICR, imprinting control region; ^b^tissue-specific parental origins of allelic expression; ^c^parental origins based on mouse studies; ^d^region known as KvDMR1.

To confirm the methylation differences using an independent approach, we performed bisulphite pyrosequencing for a subset of the novel imprinted DMRs. For this purpose, ten DMRs were selected on the basis of their low FDR (*FAM50B*, *MCCC1*, *DNAJC6*, *SORD *and *RHOBTB3*) or their biological significance to the placenta (*APC*, *DNMT1*, *IGFBP1*, *LEP *and *RASGRF1*). A high correlation between the values obtained by microarray and pyrosequencing was observed (*r *= 0.85 to 0.98; *P *< 0.0001) (Figures S2A to S2J in Additional file 1). Specifically, the DNA methylation patterns observed by pyrosequencing were concordant with those found by microarray for both (1) CpG sites analyzed by microarray and their the proximal CpG sites within the pyrosequencing assays (Figures S3A to S3J in Additional file 1) and (2) the average methylation levels of all CpG sites covered by pyrosequencing (Figures S4A to S4J in Additional file 1). DNA methylation levels of the selected loci were also assessed in sperm DNA and all were unmethylated (data not shown), suggesting they may be either secondary DMRs or maternal imprinted DMRs.

**Figure 4:**
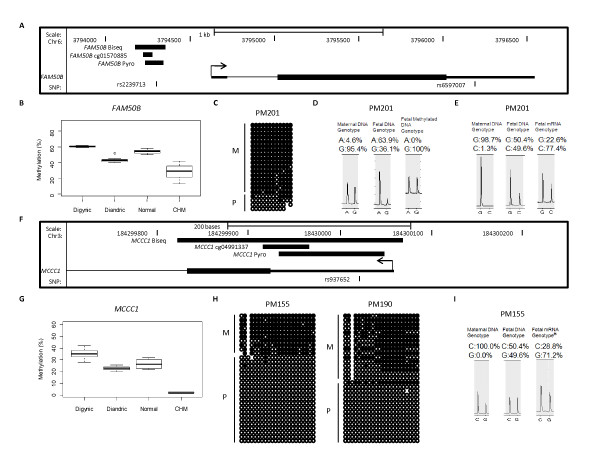
**Identification of imprinted DMRs at the proximal promoter regions of *FAM50B *and *MCCC1***. **(A) **and **(F) **Schematics showing the positions of methylation assays (Biseq: bisulphite cloning and sequencing assay; cg code: probe number of Illumina assay; and Pyro: bisulphite pyrosequencing assay) and SNP locations relative to the genes. Arrow directions represent the transcriptional directions for the genes. Genomic coordinates were retrieved from the UCSC Genome Brower database (hg18). **(B) **and **(G) **Box plots showing the methylation levels of samples from each placental group for the DMRs analyzed by bisulphite pyrosequencing. Both DMRs in *FAM50B *and *MCCC1 *have higher methylation in digynic than diandric triploid placentas, while they have intermediate methylation in normal placentas and particularly low methylation in CHMs. **(C) **and **(H) **Bisulphite cloning and sequencing showing parental origins of methylated and unmethylated alleles (M: maternal alleles; P: paternal alleles). Parental origin was determined by genotyping heterozygous informative SNPs for each sample. The DMRs in both *FAM50B *and *MCCC1 *are maternally methylated. Each black circle represents a methylated CpG dinucleotide, and each white circle represents an unmethylated CpG dinucleotide. **(D) **Quantitative genotyping of methylated alleles by pyrosequencing. SNP rs2239713 is homozygous (GG) in maternal DNA and heterozygous (AG) in foetal (placental) DNA (dispensation order: AAG). Genotyping of the placental sample using a methylation-specific pyrosequencing primer shows a homozygous (GG) pattern, indicating that the DMR associated with the maternally inherited 'G' allele is methylated while the one associated with the paternal 'A' allele is not. **(E) **and **(I) **Quantitative genotyping of expressed alleles by pyrosequencing. Both SNPs **(E) **rs6597007 (dispensation order: GGC) and **(I) **rs937652 (dispensation order for DNA genotyping: CG; dispensation order for RNA genotyping: CCG) are homozygous in maternal DNA and heterozygous in foetal DNA. Genotyping of cDNA shows a bias towards preferential expression of the paternal alleles. *The pyrosequencing primers used for cDNA genotyping (intron-spanning) in *MCCC1 *were different from those used for DNA genotyping (Table S10 in Additional file 2), so the peak ratio shown in genotyping the pyrogram of cDNA does not correspond to that for DNA.

We further evaluated DNA methylation for two genes, *FAM50B *and *MCCC1*, which contain SNPs with high average heterozygosity (about 0.4) in the proximal promoter regions that can be used to distinguish alleles (Figures 4A and 4F). Most of the other identified genes do not contain common SNPs in the nearby analyzed regions that could be used for this purpose. Bisulphite cloning and sequencing confirmed monoallelic methylation patterns for both DMRs (Figures 4C and 4H) and maternal origin of allelic methylation that was concordant with that predicted by the triploidy comparison (Figures 4B and 4G). Furthermore, allelic expression analysis showed preferential expression of the unmethylated paternal allele at the proximal promoter regions (Figures 4E and 4I), which is consistent with an inverse correlation between methylation and expression. As allelic methylation can occur in a SNP-dependent manner [27], we developed a methylation-specific pyrosequencing assay for *FAM50B *to evaluate allelic methylation in additional samples. This same approach could not be applied to *MCCC1*, because its interrogated SNP is located at a CpG site. The results of the *FAM50B *assay were concordant with cloning and sequencing results for the same placental sample (Figures 4C and 4D). As methylation was found in association with either allele (A or G at rs2239713) among 12 heterozygous normal term placental samples and ten heterozygous maternal blood samples (Table S4 in Additional file 2), the allelic methylation is not linked to the SNP genotypes, at least for this DMR.

Since diandric triploid placentas tend to be associated with trophoblast hyperplasia [17], we considered the possibility that some of the identified imprinted DMRs reflect differences in methylation between the trophoblast and mesenchyme, the two components of the chorionic villi [28]. To address this hypothesis, we used a nonimprinted, trophoblast-specific unmethylated region, *EDNRB *(Figure S5A in Additional file 1) to compare the methylation levels between diandric and digynic triploid placentas. However, we did not find a difference in methylation levels between them at this site (Figure S5B in Additional file 1). Likewise, we did not find any differences in allelic methylation between trophoblast and mesenchyme for the novel identified imprinted gene *MCCC1 *(Figures S5C and S5D in Additional file 1). However, *DNAJC6 *and *RASGRF1 *showed differential methylation between trophoblast and mesenchyme (Figure S6 in Additional file 1), which may represent cell-type-specific imprinting.

### Confirmation of parent-of-origin allelic expression for the identified putative imprinted genes

While the existence of an imprinted DMR is thought to be predictive of imprinting at the gene expression level, proving this is complicated by the fact that imprinted DMRs may exist in association with imprinted genes even in tissues where the gene is not expressed or is expressed in a biallelic manner. Furthermore, to be informative for demonstrating monoallelic expression, the placental sample must be heterozygous for an expressed SNP. To be informative for parental origin of the expressed allele, the mother must additionally be homozygous for the same SNP.

As many previously reported imprinted genes are expressed in an imprinted manner in placenta, and since we needed to screen many placenta-mother pairs to find informative cases, we proceeded to investigate the parental origin of allelic expression for the novel putative imprinted genes using a high-throughput genotyping approach, specifically the iPLEX Gold assay on the MassARRAY platform (Sequenom, Inc., San Diego, CA, USA). We selected 38 of 45 genes associated with novel imprinted DMRs (the 45 putative imprinted genes including *RASGRF1*, for which imprinted expression has not been reported in humans) on the basis of the availability of an exonic SNP with high average heterozygosity (>0.1) and the presence of expression in the placenta according to the GNF atlas database (http://biogps.gnf.org/). In addition, two exonic SNPs from *IGF2 *were included as positive controls, since *IGF2 *is well known to exhibit imprinted expression in human placentas. Thus, a total of 40 SNPs were genotyped in 27 maternal-foetal pairs, including DNA from maternal blood and the corresponding foetal normal term placenta, as well as cDNA from the same placenta.

Of these 40 SNPs, 7 did not pass the quality control criteria (<70% calls or presence of severe allelic bias) and 3 had no informative (heterozygous) genotypes in foetal DNA, leaving a total of 30 SNPs for analysis (Table S5 in Additional file 2). The two SNPs from *IGF2 *showed the expected paternal allelic expression in all informative cases (Table S5 in Additional file 2). Of the 28 novel putative imprinted genes, 11 showed monoallelic expression in at least a portion of informative samples (Table 2). Among these 11 genes, 8 had cases informative (homozygous) in maternal blood for parental origin assessment. Since most CpG sites in the microarray are located at the proximal promoter regions of the genes, we assumed that the DNA methylation would most likely correlate with silencing for all these genes. Six genes (*FAM50B*, *DNMT1*, *RHOBTB3*, *ARMC3*, *AIFM2 *and *LEP*) showed parent-of-origin-dependent expression that matched that predicted by the parental origin of the DMRs, while two others (*MOV10L1 *and *ST8SIA1*) showed parental expression opposite that predicted in one or more informative cases (Table 2). For *FAM50B *and *RHOBTB3*, monoallelic expression for both reciprocal forms of the SNP was also observed. Some genes with imprinted DMRs may not show allele-specific expression biases because of the presence of tissue-specific or gestational age-specific imprinting that is further regulated by DNA methylation at other nearby sites.

**Table 2:** Eleven genes associated with candidate imprinted DMRs with confirmed monoallelic expression^a^

Gene	DMR	SNP	Monoallelic expression, observed/total (%)	**Monoallelic expression observed for reciprocal SNP**^**b**^	**Matched expected parental origin, observed/total (%)**^**c**^
*FAM50B*	M	rs6597007	9/9 (100)	Y	5/5 (100)
*DNMT1*	M	rs16999593	1/1 (100)	-	1/1 (100)
*MOV10L1*	P	rs9617066	8/9 (89)	N	1/3 (33)
*RHOBTB3*	M	rs34896	3/4 (75)	Y	2/2 (100)
*SNCB*	M	rs2075667	3/4 (75)	N	NI
*ARMC3*	M	rs12259839	2/3 (67)	N	2/2 (100)
*ST8SIA1*	M	rs4762737	2/3 (67)	Y	0/1 (0)
*ARHGAP4*	P	rs2070097	1/2 (50)	-	NI
*AIFM2*	M	rs7908957	2/8 (25)	N	1/1 (100)
*MCCC1*	M	rs937652	2/8 (25)	Y	NI
*LEP*	P	rs2167270	1/15 (7)	-	1/1 (100)

^a^DMR, differentially methylated region; SNP, single-nucleotide polymorphism; NI, not informative. ^b^Where both alleles of SNP were observed to be expressed among cases with monoallelic expression; this is impossible to determine if only one case showed monoallelic expression. ^c^Number of cases matching the expected parental origin of those cases informative with regard to determining parent of origin.

A number of genes did not consistently show monoallelic expression using the iPLEX Gold assay. For example, for *LEP*, only 1 of 15 samples was scored as monoallelic using this approach. To evaluate the sensitivity of the iPLEX Gold genotyping assay for detecting allelic biases in expression, we developed an RNA-specific genotyping pyrosequencing assay for *LEP*. Although the two methods were correlated (*r *= 0.64; *P *< 0.02), we found that pyrosequencing was more likely to detect preferential allelic expression, with 5 of 12 informative cases exhibiting a <0.3 allelic ratio by pyrosequencing (Table S6 in Additional file 2). Furthermore, in case PM155 for *MCCC1*, we found preferential paternal allelic expression by pyrosequencing (Figure 4I), but not by iPLEX Gold genotyping (Table 2). Thus, the iPLEX Gold assay may not be sufficiently sensitive to detect more subtle allelic expression bias, that is, in circumstances where there is a mix of cells with biallelic and monoallelic expression.

### Tissue-specific and gestational age-specific methylation of imprinted DMRs

To study tissue-specific effects and the effect of gestational age on methylation of the putative imprinted DMRs, we further compared methylation at these sites among three types of foetal somatic tissues (eight brain samples, twelve kidney samples and eleven muscle samples) and two sets of placentas with different gestational ages (ten midgestation and ten term placentas) that had been run in the same Infinium HumanMethylation27 BeadChip array.

For tissue-specific methylation analysis, we compared the DNA methylation levels of the 108 DML (probes) associated with the 63 imprinted DMRs in five tissues (brain, kidney, muscle, midgestation placenta and blood). Multiclass comparison from SAM was performed with 1,000 permutations. Using a <0.1% FDR cutoff, 53 probes of 46 imprinted DMRs showed differential DNA methylation between tissues (Table 3 and Table S7 in Additional file 2). Placenta-specific methylation was observed for 31 of these probes (26 imprinted DMRs), with the average methylation being more than 15% higher in placenta than in any other tissues (Table 3 and Table S7 in Additional file 2). A change in methylation of placenta by gestational age was found for 12 probes from ten DMRs using the same statistical criterion (<0.1% FDR) (Table 3 and Table S8 in Additional file 2). Thus, imprinted DMRs can show both tissue-specific and gestational age-specific DNA methylation. Nonetheless, 14 of the imprinted DMRs showed constant methylation between different tissues and gestational ages (Table 3 and Table S9 in Additional file 2), 11 of which are in ICRs from known imprinted genes. Three identified imprinted DMRs associated with *FAM50B*, *FGF12 *and *IRF7 *also remained constant across samples and are thus potential ICRs or primary DMRs.

**Table 3:** DNA methylation of identified DMRs in different tissues and gestational ages^a^

Index	Gene	Chromosome	**Tissue-specific**^**b**^	**Change in gestation**^**c**^	**Stable non-tissue-specific**^**d**^	**Known imprinted genes**^**e**^
1	*DNAJC6*	1	Y^f^	Y	N	N
2	*LASS2*	1	Y^f^	Y	N	N
3	*PEX5*	12	Y^f^	Y	N	N
4	*RASGRF1*	15	Y^f^	N	N	N
5	*AKAP10*	17	Y^f^	N	N	N
6	*AIFM2*	10	Y^f^	N	N	N
7	*APC*	5	Y^f^	N	N	N
8	*ARHGAP4*	X	Y^f^	N	N	N
9	*ARMC3*	10	Y^f^	N	N	N
10	*C3orf62*	3	Y^f^	N	N	N
11	*CD83*	6	Y^f^	N	N	N
12	*CMTM3*	16	Y^f^	N	N	N
13	*DNMT1*	19	Y^f^	N	N	N
14	*G0S2*	1	Y^f^	N	N	N
15	*GATA4*	8	Y^f^	N	N	N
16	*LEP*	7	Y^f^	N	N	N
17	*MCCC1*	3	Y^f^	N	N	N
18	*NUDT12*	5	Y^f^	N	N	N
19	*PCK2*	14	Y^f^	N	N	N
20	*RHOBTB3*	5	Y^f^	N	N	N
21	*SLC46A2*	9	Y^f^	N	N	N
22	*SNCB*	5	Y^f^	N	N	N
23	*SORD*	15	Y^f^	N	N	N
24	*ST8SIA1*	12	Y^f^	N	N	N
25	*TBX6*	16	Y^f^	N	N	N
26	*TMEM17*	2	Y^f^	N	N	N
27	*ZNF232*	17	Y^f^	N	N	N
28	*ZNF396*	18	Y^f^	N	N	N
29	*AK094715*	6	Y	Y	N	N
30	*DIRAS3*	1	Y	Y	N	Y
31	*CMTM8*	3	Y	Y	N	N
32	*SEMA3B*	3	Y	Y	N	N
33	*CDKN1C*	11	Y	N	N	Y
34	*H19*	11	Y	N	N	Y
35	*KCNQ1*	11	Y	N	N	Y
36	*MEG3*	14	Y	N	N	Y
37	*PEG10*	7	Y	N	N	Y
38	*C10orf125*	10	Y	N	N	N
39	*CCR10*	17	Y	N	N	N
40	*CYP2W1*	7	Y	N	N	N
41	*FIGNL1*	7	Y	N	N	N
42	*IGFBP1*	7	Y	N	N	N
43	*MOV10L1*	22	Y	N	N	N
44	*P2RY6*	11	Y	N	N	N
45	*PARP12*	7	Y	N	N	N
46	*SAMD10*	20	Y	N	N	N
47	*L3MBTL*	20	N	Y	N	Y
48	*ACPL2*	3	N	Y	N	N
49	*REEP6*	19	N	Y	N	N
50	*GNAS*(M)	20	N	N	Y	Y
51	*GNAS*(P)	20	N	N	Y	Y
52	*GRB10*	7	N	N	Y	Y
53	*MEST*	7	N	N	Y	Y
54	*NAP1L5*	4	N	N	Y	Y
55	*PEG3*	19	N	N	Y	Y
56	*PLAGL1*	6	N	N	Y	Y
57	*SGCE*	7	N	N	Y	Y
58	*SNURF*	15	N	N	Y	Y
59	*ZIM2*	19	N	N	Y	Y
60	*ZNF597*	16	N	N	Y	Y
61	*FAM50B*	6	N	N	Y	N
62	*FGF12*	3	N	N	Y	N
63	*IRF7*	11	N	N	Y	N

^a^DMR, differentially methylated region; ^b^multiclass comparison of methylation level in brain, kidney, muscle, midgestation placenta and blood with FDR <0.1%; ^c^Multiclass comparison of methylation level in early-gestation, midgestation and term placenta, FDR <0.1%; ^d^DMRs with no statistically significant changes in methylation level in different tissues and gestational ages; ^e^Based on the public databases (http://igc.otago.ac.nz/ and http://www.geneimprint.com/); ^f^placenta-specific methylation.

**Figure 5:**
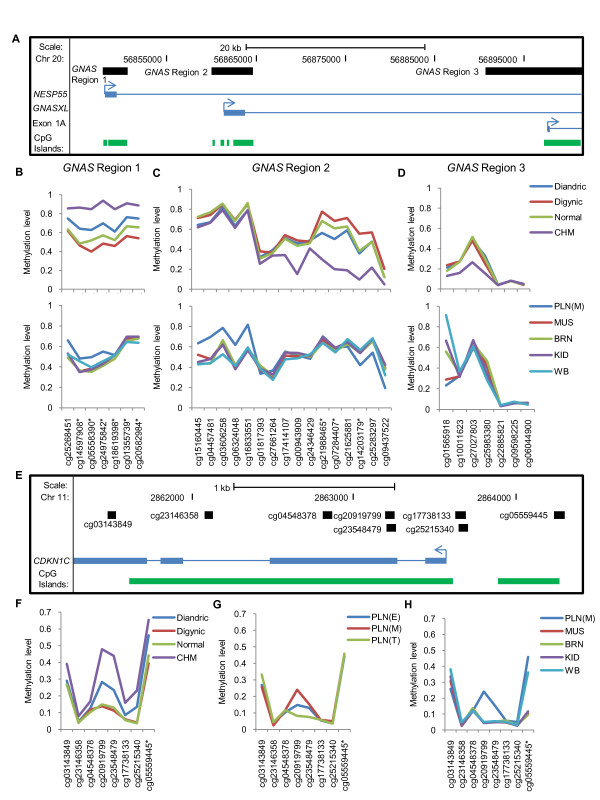
**Illustration of tissue-specific and gestational age-specific methylation at the proximal promoter regions of *GNAS *and *CDKN1C***. **(A) **and **(E) **Schematics showing the positions of the Illumina Infinium probes relative to the genes and transcripts. Arrow directions represent the transcriptional directions for the genes or transcripts. Genomic coordinates were retrieved from the UCSC Genome Brower database (hg18). **(B) **through **(D) **Average methylation levels of the Illumina Infinium probes in different placental groups (top) and in different tissues (bottom). Probe numbers are shown on the *x*-axes in the bottom panels divided into **(B) ***GNAS *region 1, **(C) ***GNAS *region 2 and **(D) ***GNAS *region 3 according to their proximity to the known transcripts. Tissue-specific methylation can be found from cg15160445 to cg16833551 in *GNAS *region 2 and at cg01565918 in *GNAS *region 3. **(F) **through **(H) **Average methylation levels of the Illumina Infinium probes of *CDKN1C *in **(F) **different placental groups, **(G) **different gestational ages of placenta and **(H) **different tissues. Probe numbers are shown on the *x*-axes. Both tissue-specific and gestational age-specific methylation can be found at cg20919799. PLN(E): early gestation placenta; PLN(M): midgestation placenta; PLN(T): term placenta; MUS: muscle; BRN: brain; KID: kidney; WB: whole blood.

The complexity of DNA methylation associated with imprinted genes can be illustrated by the data for three known imprinted genes, *GNAS*, *CDKN1C *and *MEST*, for which multiple probes were present on the Infinium HumanMethylation27 BeadChip array. For *GNAS*, the array contains probes for 30 CpG sites mapping across three promoter regions of three alternative transcripts (*NESP55*, *GNASXL *and exon 1A of *GNAS*) (Figure 5A). As previously reported, the paternal DMR is located at the promoter of *NESP55 *transcript (Figure 5B), while the maternal DMR is located at the promoter of *GNASXL *[29]. While most of the CpG sites have more or less equal average methylation across the locus, cg15160445 to cg1683351 and cg01565918 show clear tissue-specific methylation across different tissues (Figures 5B to 5D). For *CDKN1C*, there are eight probes present in the array (Figure 5E). A previously unidentified paternal DMR was identified at the promoter region of this gene through our comparison of triploids (Figure 5F). Interestingly, not only is the imprinted DMR itself tissue-specific (that is, it is a secondary DMR) (Table 3) but there is also a probe (cg20919799) that shows differential methylation across different gestational ages (Figure 5G) and tissues (Figure 5H). Likewise, for *MEST*, for which ten probes span two regions of the gene (Figure S7A in Additional file 1), an imprinted DMR can be found in one region (Figures S7B and S7C in Additional file 1), while tissue-specific and gestational age-specific methylation is observed in another region of the *MEST *promoter (Figures S7C to S7G in Additional file 1).

## Discussion

Many efforts have been made to identify imprinted genes in the human genome because of their importance in foetal growth and development and their potential for dysregulation [11,12]. Most known imprinted genes to date were first identified in mice, but many imprinted genes are not conserved across species [5]. In the present study, we utilized diandric and digynic triploid placentas to map imprinted DMRs, sites that are typically associated with imprinted genes, in the human genome. We identified 11 of the 18 previously reported human ICRs covered by the Illumina Infinium HumanMethylation27 BeadChip panel, with additional ones which showed differences that were insufficient to reach our stringent statistical criteria. Furthermore, we confirmed the parent-of-origin dependence of methylation and expression in a subset of our candidate novel imprinted genes on the basis of independent experiments.

This approach improves upon previous strategies for mapping imprinted DMRs, such as comparing parthenogenotes (ovarian teratomas) and androgenotes (CHMs) [13,14], which is limited by the grossly abnormal nature of these samples, or comparing maternal and paternal uniparental disomies (UPDs) [30,31], which is restricted by the rarity of UPDs for many chromosomes and the limited tissues available for analysis. Although triploid placentas do exhibit some abnormal pathology, their cellular composition is comparable and methylation profiles of both types of triploidy were closely correlated with chromosomally normal placentas (*r *= 0.99). In comparison, a previous study showed that mature ovarian teratomas have a methylation profile more similar to that of blood (*r *= 0.94) than to either CHMs (*r *= 0.84) or normal placentas (*r *= 0.88) [14]. Genome-wide transcriptome analysis has also been used to identify imprinted genes [11,32], but it is gene expression- and SNP-dependent; thus, imprinted genes with tissue-specific expression or lacking a heterozygous exonic SNP would be missed.

As demonstrated, tissue-specific methylation of imprinted DMRs or their flanking regions can readily be assessed by comparing methylation profiles of a variety of tissues, allowing a comprehensive analysis of tissue-specific methylation regulation at complex loci, such as *GNAS *[29]. The regional dependent methylation patterns in the promoters of imprinted genes show the importance of locating the specific CpG sites defining the imprinted DMRs when studying the dynamics of promoter DNA methylation at such genes. While in the present study we identified only loci that demonstrated parent-of-origin-dependent differential DNA methylation in placenta, most known imprinted genes show parent-of-origin-specific expression in this organ [2]. Furthermore, as diandric and digynic triploids can both exist as foetuses, additional comparisons could be used to identify any potential genes that exhibit imprinting specifically in other tissues. This study is limited by the low coverage of CpG sites in the array (about two CpG sites on average for each proximal promoter region of genes), which reduces its power to identify imprinted DMRs as these may be limited to specific regions within the promoter. This analysis could thus be extended further by using microarray or whole-genome sequencing with greater coverage of the genome.

Overall, the number of imprinted DMRs identified in the present study was less than that predicted by bioinformatics approaches [33]. However, the stringent selection criteria (<0.1% FDR and absolute average methylation difference >15%) that we used to pick the top candidate sites caused an underestimation of the number of imprinted loci. Many more candidate imprinted DMRs can be identified with this data set using lower thresholds. In fact, many known imprinted genes that we failed to identify on the basis of these criteria did show nominally significant (*P *< 0.05 without correction for multiple comparisons) DNA methylation differences between diandric and digynic triploids (Table S3 in Additional file 2). For instance, a recently confirmed imprinted gene, *RB1 *[34], was significantly differentially methylated between diandric and digynic triploidies (<0.1% FDR), with a methylation pattern consistent with that of a maternal DMR (data not shown). However, it was excluded because its absolute average methylation difference between diandries and digynies was only 14%. While we expect imprinted DMRs to show a difference of 33% between the triploid groups, smaller differences may be observed owing to a lack of complete methylation at all CpG sites on the inactive allele or to the presence of a mix of cell types, only some of which are imprinted. Similarly, methylation on the inactive X chromosome in females is incomplete (much less than 50%) in the placenta for gene promoter regions that are typically methylated at 50% in somatic tissues, despite still showing a significant increase in methylation relative to male placenta [22].

Only some of the novel putative imprinted DMRs could be confirmed to show monoallelic expression, and others did not show strict parent-of-origin expression in all placentas (Table S5 in Additional file 2). There are several possible explanations. First, there may be cell- or tissue-specific imprinting confounding the ability to detect a difference in whole villous samples from term placentas. Many known imprinted genes show imprinted expression only in specific placental cell types, for example, *Mash2 *in mice, which is differentially expressed only in diploid trophoblast cells of the postimplantation embryo [35], and *STOX1 *in humans, which is maternally expressed in extravillous trophoblast cells [36]. Given the highly heterogeneous cell types present in the placenta [28], nonimprinted expression in some cells may mask allelic expression in others. The possibility that cell heterogeneity exists for the DMRs identified in the present study is supported by the observations that (1) average methylation of some candidate DMRs was not the expected 50% in normal placentas (Figure S3 in Additional file 1) and (2) *DNAJC6 *and *RASGRF1 *showed differential methylation between trophoblast and mesenchyme (Figure S6 in Additional file 1). Second, as we have shown, the iPLEX Gold assay may not be sensitive enough to pick up subtle allelic expression biases (Table S6 in Additional file 2).

Third, there may be alternative transcripts regulated by alternative promoters that are not imprinted, so the observed expressed allelic ratio at particular SNPs may be complicated by the synergic effect of multiple transcripts. Such complex regulation is observed for known imprinted genes such as *GNAS*, *CDKN1C *and *MEST *(Figure 5 and Figure S5 in Additional file 1). However, allelic expression from either parent in some genes, such as *MOV10L1 *and *ST8SIA1*, suggests that some of the identified DMRs may be random monoallelically expressed genes instead of imprinted genes (with DNA methylation differences between diandries and digynies occurring by chance).

The validation of all the putative imprinted DMRs we identified is limited by the number of samples and common SNPs within regions and by the availability of intact mRNA from the pathological specimens. A proper validation experiment to demonstrate that the DMRs we have identified are associated with imprinted methylation and gene expression requires being able to trace the parental origin of the methylated and expressed alleles in multiple members of the same family, which can be done in mice but is impractical and ethically impossible to do across multiple tissues in humans [37]. The best alternative is to trace the origin of the methylated allele and the expressed allele in multiple individuals. This requires a SNP adjacent to the methylation site that is heterozygous in the test sample but homozygous in one parent. Using this strategy, we demonstrated for *FAM50B *that (1) a maternal origin of the methylated allele in placenta and blood from multiple individuals and on reciprocal genetic backgrounds and (2) the paternal allele is expressed with either SNP allele in the placenta, thus ruling out the possibility of a genetic effect. Confirming that an imprint represents a primary imprinted DMR requires detailed investigations of postfertilization imprinting dynamics, which is difficult to perform in humans. Nonetheless, we showed that the methylation level of *FAM50B *is similar in multiple tissues and is unmethylated in sperm, suggesting that it is likely to be a primary maternal DMR. During the revision of this manuscript, the maternal imprint of *FAM50B *was also confirmed by other groups using similar validation methods [38,39]. The goal of the present study was to demonstrate the ability of our approach to identify imprinted DMRs, not to map and confirm every imprinted DMR on the array. Thus, the putative imprinted DMRs listed in the present study should be considered with caution, and further validation is required.

Two genes identified as potentially being imprinted in the present study, *APC *and *DNMT1*, were excluded as being imprinted in previous studies [40,41], while *APC *was reported as being imprinted in another study [42]. Of interest, *DNMT1 *is a DNA methyltransferase that is important for the maintenance and establishment of DMRs in imprinted genes [43], while *APC *is a negative regulator of the Wnt signalling pathway, which has been implicated in the survival, differentiation and invasion of human trophoblasts [40]. Although *Dnmt1 *was found to be dispensable for growth of the extraembryonic lineages in mice [44], it is not methylated at the orthologous region in mice [41]. Both the *APC *and *DNMT1 *DMRs were reported to be specifically methylated in primate placentas [45], suggesting that the potential imprinting marks of these genes emerged fairly recently in evolution. This is also consistent with the hypothesis that maternal imprints are under selective pressure during early development for methylation-dependent control [46]. This could occur by selecting genes with developmental advantage by gain of imprinting from epipolymorphisms [47].

## Conclusions

In conclusion, we have demonstrated that comparison of diandric and digynic triploids is an effective method for mapping imprinted DMRs in the human genome. This approach can be extended to different tissues, gestational ages or species, thereby generating a comprehensive view of imprinting regulation and evolution. The ability to map novel imprinted DMRs in the human genome should improve our understanding of the causes of placental dysfunction and birth defects. With the rapid advancement of molecular genetics technologies, a complete map of imprinted DMRs may ultimately be generated by the use of whole-genome sequencing. However, the present approach is a convenient, currently available and cost-effective method of imprinted gene mapping.

## Methods

### Sample collection

This study was approved by the ethics committees of the University of British Columbia and the Children's & Women's Health Centre of British Columbia. Early gestation placental samples (ten diandric triploids, ten digynic triploids, six CHMs and ten normal controls) were obtained from spontaneous abortions examined in the Children's & Women's Health Centre of British Columbia pathology laboratory. The parental origin of triploids was determined by using microsatellite polymorphisms as previously described [17-19], and these studies also allowed us to exclude maternal contamination in the placental samples. Midgestation placental samples (*n *= 10) and foetal tissues (11 muscle samples, 12 kidney samples and 8 brain samples) were obtained from anonymous, chromosomally normal, second-trimester elective terminations for medical reasons. Term placental samples and the corresponding maternal blood samples were collected from Children's & Women's Health Centre of British Columbia with the women's written informed consent. For all placental samples, fragments of about 1 cm^3 ^were dissected from the foetal side and whole villi were used for investigation. All tissues were karyotyped for chromosomal abnormalities, and genomic DNA was extracted from each tissue sample using standard techniques. Total RNA was extracted from term placentas using an RNeasy kit (Qiagen, Valencia, CA, USA) according to the manufacturer's instructions.

### Illumina DNA methylation array

Genomic DNA was bisulphite-converted using the EZ DNA Methylation Kit (Zymo Research, Orange, CA, USA) according to the manufacturer's instructions. Bisulphite treatment converted unmethylated cytosines to uracils while leaving methylated cytosines unchanged. After DNA purification, bisulphite-converted DNA samples were randomly arrayed and subjected to the Infinium HumanMethylation27 BeadChip panel array-based assay. The array assays methylation levels at 27,578 CpG sites in the human genome. The methylation level for each CpG site was measured by the intensity of fluorescent signals corresponding to the methylated allele (Cy5) and the unmethylated allele (Cy3). Cy5 and Cy3 fluorescence intensities were corrected independently for background signal and normalized using GenomeStudio software (Illumina, Inc.). Continuous β values that range from 0 (unmethylated) to 1 (methylated) were used to identify the percentage of methylation, from 0% to 100%, for each CpG site. The β value was calculated based on the ratio of methylated/(methylated + unmethylated) signal outputs. The detection *P *value of each probe was generated by comparison with a series of negative controls embedded in the assay. Probes with detection *P *values >0.05 in any of the samples were eliminated from the study. The correlation coefficient for technical replicates was >0.98. The microarray data from this study have been submitted to the NCBI Gene Expression Omnibus (http://www.ncbi.nlm.nih.gov/geo) under accession number GSE25966.

### DNA methylation analyses for targeted loci

Methylation-unbiased PCR and sequencing primers were designed based on the probe sequences provided by Illumina (Table S10 in Additional file 2). All primers were designed in regions free of known SNPs. Pyrosequencing was performed using a PyroMark MD system (Biotage, Uppsala, Sweden). The quantitative levels of methylation for each CpG dinucleotide were evaluated using Pyro Q-CpG software (Biotage). For bisulphite cloning and sequencing, the PCR product from individual samples was generated by using non-biotinylated primers (Table S10 in Additional file 2) and subsequently TA-cloned into the pGEM-T Easy Vector System (Promega, Madison, WI, USA). Individual clones were picked and PCR-amplified with SP6 and T7 promoter primers. PCR products were sequenced by using Sanger sequencing. The sequencing data were analyzed using BiQ Analyzer Software [48], and sequences with less than an 80% bisulphite conversion rate were eliminated from analysis.

### SNP genotyping

Multiplex genotyping of genomic DNA and cDNA was performed by using the iPLEX Gold assay on the MassARRAY platform (Sequenom) at the Génome Québec Innovation Centre (Montréal, PQ, Canada). Primers for SNP genotyping were designed by using primer design software from Sequenom (Table S11 in Additional file 2). The primer extended products were analyzed and the genotypes were determined by mass spectrometric detection using the MassARRAY Compact System (Sequenom). Technical replicates showed a correlation of *r *= 0.92. Samples or SNPs with <70% conversion rates (calls) were eliminated. Genotyping by pyrosequencing was performed on a PyroMark MD System, and the relative levels of alleles for SNPs were evaluated by using PSQ 96MA SNP software (Biotage). Genotyping of exonic SNPs was carried out with cDNA prepared using either (1) the Omniscript Reverse Transcriptase Kit (Qiagen) followed by the iPLEX Gold assay or pyrosequencing or (2) the Qiagen OneStep RT-PCR Kit followed by pyrosequencing. Primers for pyrosequencing genotyping were designed by using primer design software from Biotage (Table S11 in Additional file 2). PCR without reverse transcriptase was performed on each sample to confirm that there was no genomic DNA contamination.

### Statistical analysis

Unsupervised hierarchical clustering of samples was done using Illumina GenomeStudio software. Differentially methylated probes in the Illumina Infinium HumanMethylation27 BeadChip array from each comparison were identified using the siggenes package from R software with a cutoff of <0.1% FDR. FDRs were generated after comparison of 1,000 random permutations between samples. The Pearson linear correlation coefficient was used to determine the similarity of DNA methylation profiles between samples. The Database for Annotation, Visualization and Integrated Discovery (DAVID) program was used for gene ontology analysis using the total number of genes presented in the array as a background for comparison [49,50].

## Abbreviations

CHM: complete hydatidiform mole; CNV: copy number variation; DML: differentially methylated loci; DMR: differentially methylated region; FDR: false discovery rate; GO: Gene Ontology; ICR: imprinting control region; PCR: polymerase chain reaction; SAM: Significance Analysis of Microarrays; SNP: single-nucleotide polymorphism.

## Competing interests

The authors declare that they have no competing interests.

## Authors' contributions

RKCY and WPR conceived the study. RKCY designed and performed the experiments. RJ prepared and karyotyped the samples. MSP performed the microarray experiment. RKCY analyzed the data. DEM contributed the tissue samples. RKCY and WPR wrote the paper. All authors read and approved the final manuscript.

## Supplementary Material

Additional file 1 **Figures S1 to S6**. Click here for file

Additional file 2 **Tables S1 to S11**. Click here for file
