# Supplementary material for: Genome-wide mapping of imprinted differentially methylated regions by DNA methylation profiling of human placentas from triploidies
Source: Epigenetics Chromatin. 2011 Jul 13;4:10. doi: 10.1186/1756-8935-4-10 (PMC3154142; doi:10.1186/1756-8935-4-10)
Supplement: Additional file 1 — Figures S1 to S6. [file 1756-8935-4-10-S1.PDF]

Supplemental Figure Legends

Figure S1 - Analyses of DNA methylation data from the Illumina microarray assay

(A) Unsupervised hierarchical clustering of placental samples. No preferential clustering by gender is observed. Sample names are shown with labelling of corresponding tissue types. Samples were clustered by hierarchical clustering of beta values based on 1-r (Illumina Genome studio software), where r is referring to the correlation coefficient between samples. Digynic triploids are indicated with grey boxes, diandric triploids are indicated with black boxes and normal placentas are indicated with white boxes. Female placentas are labelled in red while male placentas are labelled in blue. (B) Pair-wise comparisons of average methylation of probes between different placental groups. Scatterplots of average methylation of probes between placental pairs are shown on the upper right panel while their correlation coefficients are shown on the lower left panel. Density plots of the methylation distribution of probes in each placental group are shown between two panels. AvgA: average methylation in diandric triploids, AvgG: average methylation in digynic triploids, AvgN: average methylation in chromosomally normal placentas, AvgC: average methylation in CHMs and AvgB: average methylation in blood samples. (C) Distribution of p values calculated by the Student's t test. More than 2000 probes have p values lower than 0.01. (D) Scatterplot of methylation values for identified DML in all digynic vs. diandric triploid samples. The DNA methylation level for comparisons of all samples is given with the maternal DML represented by red circles and paternal DML represented by blue circles. Maternal DML and paternal DML form two independent clusters without much overlap. DML: differentially methylated loci. (E) Scatterplot of average methylation for each maternal DML and paternal DML for each pairwise comparison of placental groups. Scatterplots for each comparison is shown on the lower right panel while the corresponding correlation

coefficients are shown on the upper left panel. Average methylation of maternal DML is highlighted in pink while average methylation of paternal DML is highlighted in light blue.

Figure S2 - Correlation of DNA methylation measurements between the Illumina array and pyrosequencing

Methylation level measured by Illumina array (beta-value) for all the placental samples are compared against estimated percent methylation of the same CpG sites measured by pyrosequencing for (A) *APC*, (B) *DNAJC6*, (C) *DNMT1*, (D) *FAM50B*, (E) *IGFBP1*, (F) *LEP*, (G) *MCCC1*, (H) *RASGRF1*, (I) *RHOBTB3* and (J) *SORD*.

Figure S3 - DNA methylation patterns of all CpG sites measured within each individual pyrosequencing assay

Methylation levels measured by pyrosequencing are shown for (A) *APC*, (B) *DNAJC6*, (C) *DNMT1*, (D) *FAM50B*, (E) *IGFBP1*, (F) *LEP*, (G) *MCCC1*, (H) *RASGRF1*, (I) *RHOBTB3* and (J) *SORD*. CpG numbers are assigned according to the ascending order of CpG sites covered by the pyrosequencing assay. CpG sites with an asterisk are the sites targeted by probes on the Illumina array. Values observed for each sample are indicated by coloured dots corresponding to the placental group, while lines connect the group averages at each site.

Figure S4 - Comparison of average DNA methylation level of identified imprinted DMRs between placental groups

Boxplots of average methylation in each placental group are shown for (A) *APC*, (B) *DNAJC6*, (C) *DNMT1*, (D) *FAM50B*, (E) *IGFBP1*, (F) *LEP*, (G) *MCCC1*, (H) *RASGRF1*, (I) *RHOBTB3* and (J) *SORD*.

Figure S5 - Evaluation of cell composition as potential confounders to the imprinted DMR identification approach

(A) Methylation level at the promoter region of *EDNRB* is used as a trophoblast marker as it has low methylation in trophoblast and is more highly methylated in mesenchymal cells. Ratio of trophoblasts to mesenchyme cells can be estimated by measuring the methylation level in the placenta. (B) *EDNRB* shows no differential methylation (i.e. no difference in trophoblasts to mesenchymal cell ratio) between digynic and diandric triploid placentas. (C,D) Parent-of-origin dependent allelic methylation of *MCCC1* can be found in both (C) trophoblasts and (D) mesenchymal cells.

Figure S6 - Evaluation of cell-type specific DNA methylation of identified imprinted DMRs

Methylation levels measured by pyrosequencing are shown for (A) *APC*, (B) *DNAJC6*, (C) *DNMT1*, (D) *FAM50B*, (E) *IGFBP1*, (F) *LEP*, (G) *MCCC1*, (H) *RASGRF1*, (I) *RHOBTB3* and (J) *SORD* in trophoblast and mesenchyme. Cell-type specific methylation can be found in *DNAJC6* and *RASGRF1*.

Figure S6 - Illustration of tissue-specific and gestational age-specific methylation at the proximal promoter regions of *MEST*

(A) Schematic diagram shows the positions of probes contained on the Illumina Infinium methylation array relative to the transcripts. The directions of arrows represent the transcriptional directions. Genomic coordinates are retrieved from the UCSC Genome Browser (hg18). (B-G) Comparison of average methylation level of the Illumina Infinium probes between: (B and C) different placental groups; (D and E) placentas with different gestational ages; (F and G) different tissues. Probe numbers are shown on the x-axis of the figures in the lower panel divided

into (B, D, F) *MEST* Region 1 and (C, E, G) *MEST* Region 2 according to their proximity to the known transcripts. PLN(E): early gestation placenta, PLN(M): mid gestation placenta, PLN(T): term placenta, MUS: muscle, BRN: brain, KID: kidney and WB: whole blood.

Figure S1

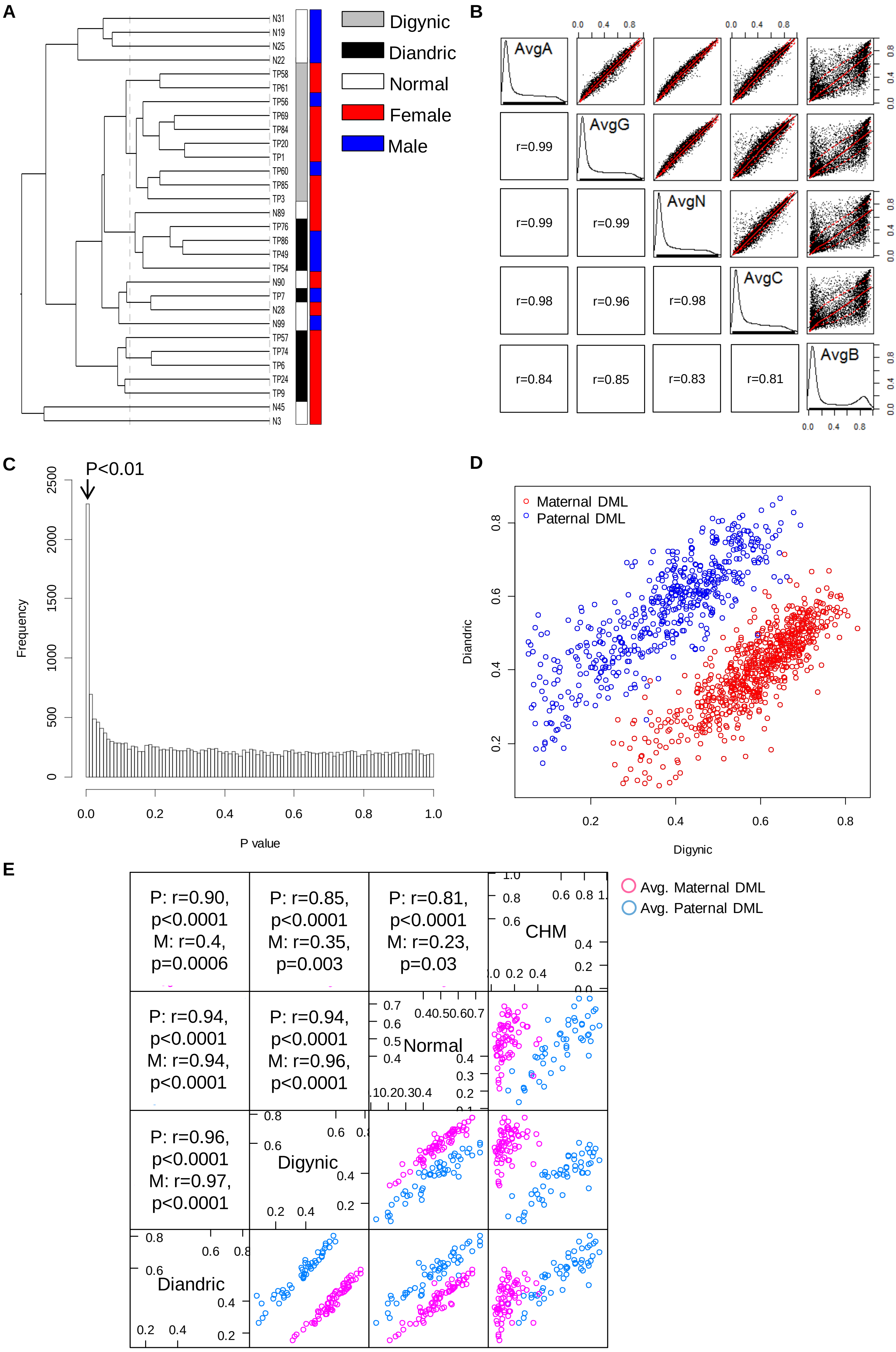
A
N31
N19
N25
N22
TP58
TP61
TP56
TP69
TP84
TP20
TP1
TP60
TP85
TP3
N89
TP76
TP86
TP49
TP54
N90
TP7
N28
N99
TP57
TP74
TP6
TP24
TP9
N45
N3
Digynic
Diandric
Normal
Female
Male
B
AvgA
AvgG
AvgN
AvgC
AvgB
r=0.99
r=0.99
r=0.99
r=0.98
r=0.96
r=0.98
r=0.84
r=0.85
r=0.83
r=0.81
C
P<0.01
Frequency
P value
D
Maternal DML
Paternal DML
Diandric
Digynic
E
P: r=0.90, p<0.0001 M: r=0.4, p=0.0006
P: r=0.85, p<0.0001 M: r=0.35, p=0.003
P: r=0.81, p<0.0001 M: r=0.23, p=0.03
CHM
P: r=0.94, p<0.0001 M: r=0.94, p<0.0001
P: r=0.94, p<0.0001 M: r=0.96, p<0.0001
Normal
P: r=0.96, p<0.0001 M: r=0.97, p<0.0001
Digynic
Diandric
Avg. Maternal DML
Avg. Paternal DML

Figure S2

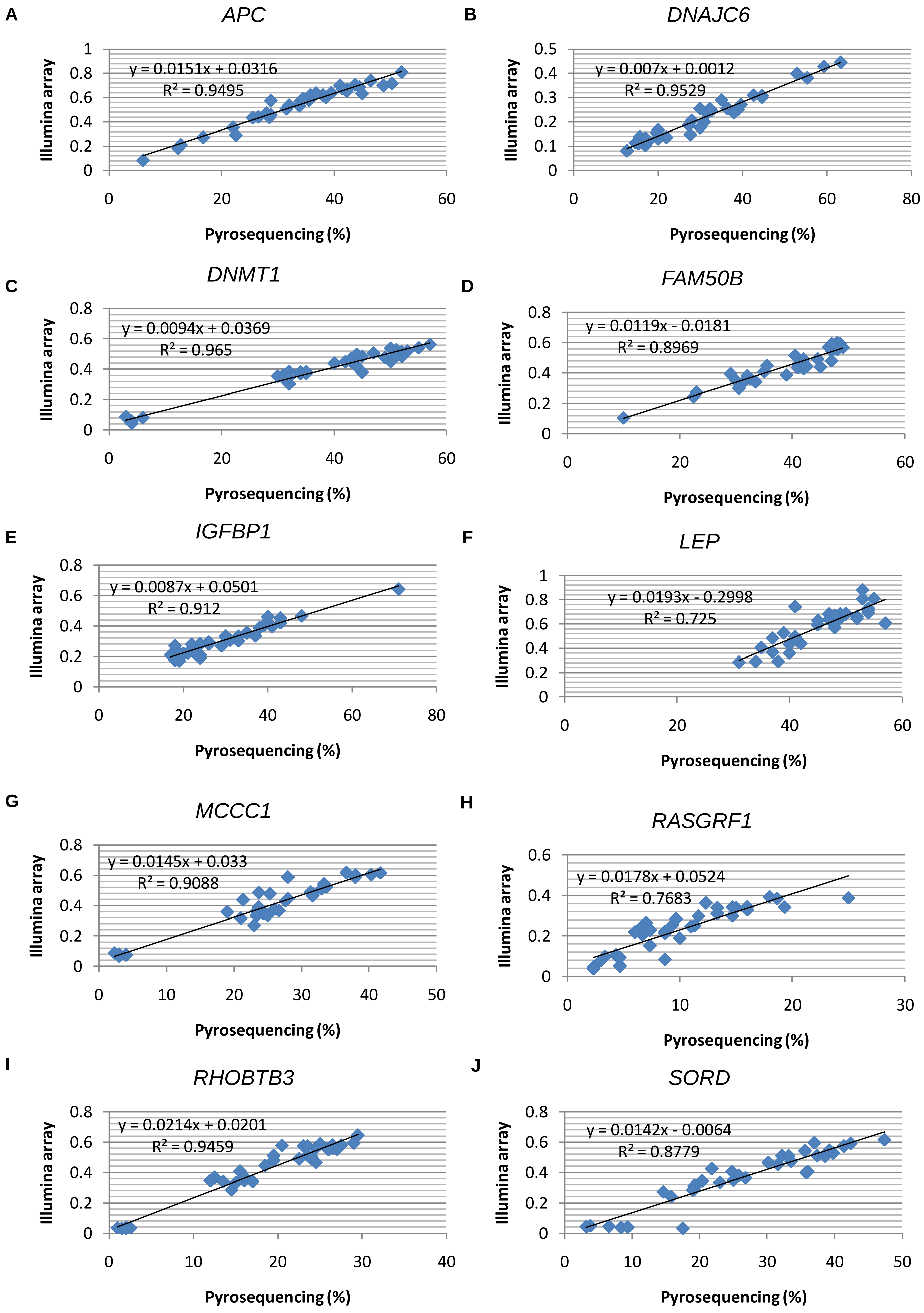
A
APC
y = 0.0151x + 0.0316
R² = 0.9495
Illumina array
Pyrosequencing (%)
0 0.2 0.4 0.6 0.8 1
0 20 40 60
B
DNAJC6
y = 0.007x + 0.0012
R² = 0.9529
Illumina array
Pyrosequencing (%)
0 0.1 0.2 0.3 0.4 0.5
0 20 40 60 80
C
DNMT1
y = 0.0094x + 0.0369
R² = 0.965
Illumina array
Pyrosequencing (%)
0 0.2 0.4 0.6 0.8
0 20 40 60
D
FAM50B
y = 0.0119x - 0.0181
R² = 0.8969
Illumina array
Pyrosequencing (%)
0 0.2 0.4 0.6 0.8
0 20 40 60
E
IGFBP1
y = 0.0087x + 0.0501
R² = 0.912
Illumina array
Pyrosequencing (%)
0 0.2 0.4 0.6 0.8
0 20 40 60 80
F
LEP
y = 0.0193x - 0.2998
R² = 0.725
Illumina array
Pyrosequencing (%)
0 0.2 0.4 0.6 0.8 1
0 20 40 60
G
MCCC1
y = 0.0145x + 0.033
R² = 0.9088
Illumina array
Pyrosequencing (%)
0 0.2 0.4 0.6 0.8
0 10 20 30 40 50
H
RASGRF1
y = 0.0178x + 0.0524
R² = 0.7683
Illumina array
Pyrosequencing (%)
0 0.2 0.4 0.6
0 10 20 30
I
RHOBTB3
y = 0.0214x + 0.0201
R² = 0.9459
Illumina array
Pyrosequencing (%)
0 0.2 0.4 0.6 0.8
0 10 20 30 40
J
SORD
y = 0.0142x - 0.0064
R² = 0.8779
Illumina array
Pyrosequencing (%)
0 0.2 0.4 0.6 0.8
0 10 20 30 40 50

Figure S3

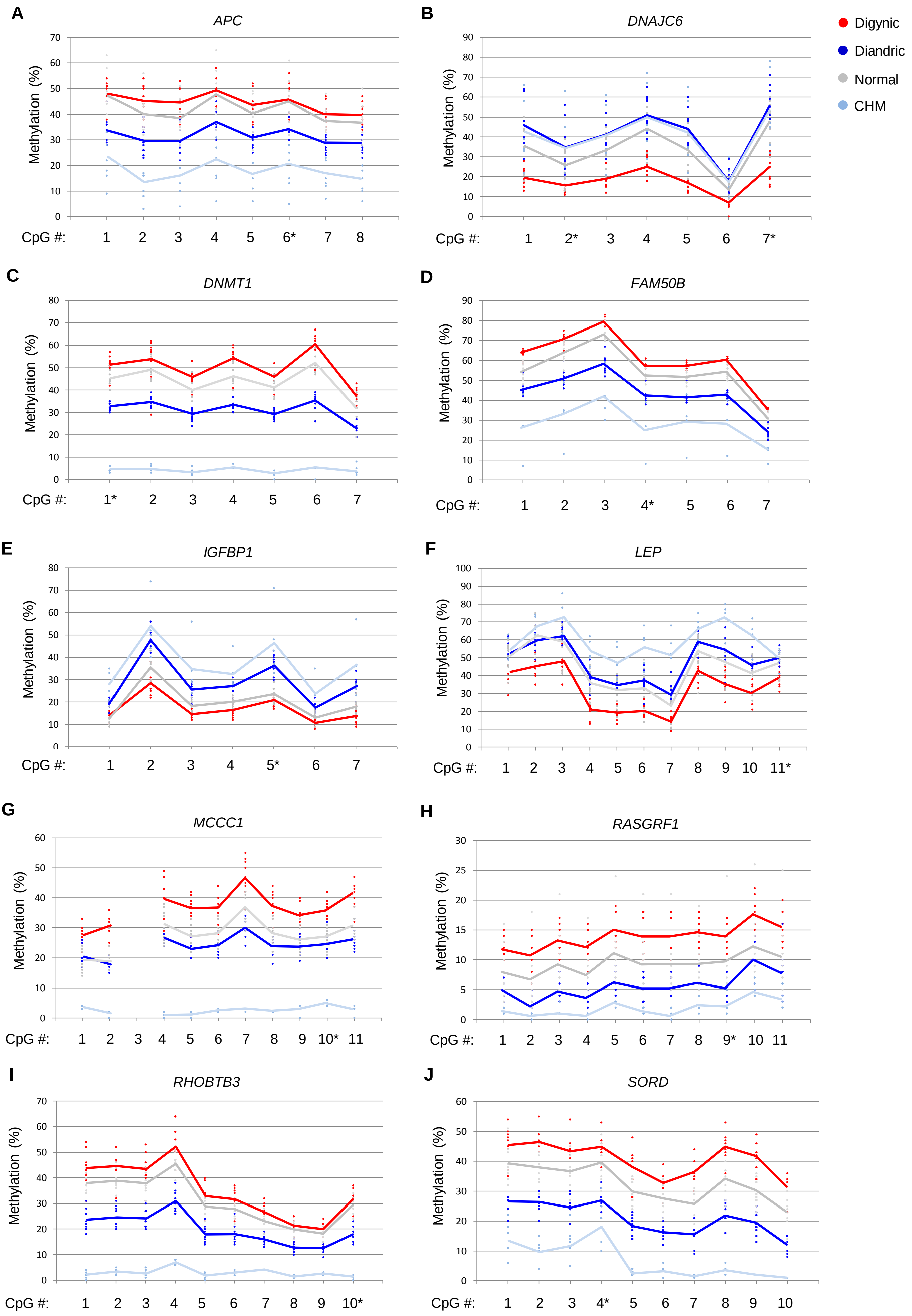

Digynic
Diandric
Normal
CHM
A
APC
Methylation (%)
0 10 20 30 40 50 60 70
CpG #: 1 2 3 4 5 6* 7 8
B
DNAJC6
Methylation (%)
0 10 20 30 40 50 60 70 80 90
CpG #: 1 2* 3 4 5 6 7*
C
DNMT1
Methylation (%)
0 10 20 30 40 50 60 70 80
CpG #: 1* 2 3 4 5 6 7
D
FAM50B
Methylation (%)
0 10 20 30 40 50 60 70 80 90
CpG #: 1 2 3 4* 5 6 7
E
IGFBP1
Methylation (%)
0 10 20 30 40 50 60 70 80
CpG #: 1 2 3 4 5* 6 7
F
LEP
Methylation (%)
0 10 20 30 40 50 60 70 80 90 100
CpG #: 1 2 3 4 5 6 7 8 9 10 11*
G
MCCC1
Methylation (%)
0 10 20 30 40 50 60
CpG #: 1 2 3 4 5 6 7 8 9 10* 11
H
RASGRF1
Methylation (%)
0 5 10 15 20 25 30
CpG #: 1 2 3 4 5 6 7 8 9* 10 11
I
RHOBTB3
Methylation (%)
0 10 20 30 40 50 60 70
CpG #: 1 2 3 4 5 6 7 8 9 10*
J
SORD
Methylation (%)
0 10 20 30 40 50 60
CpG #: 1 2 3 4* 5 6 7 8 9 10

Figure S4

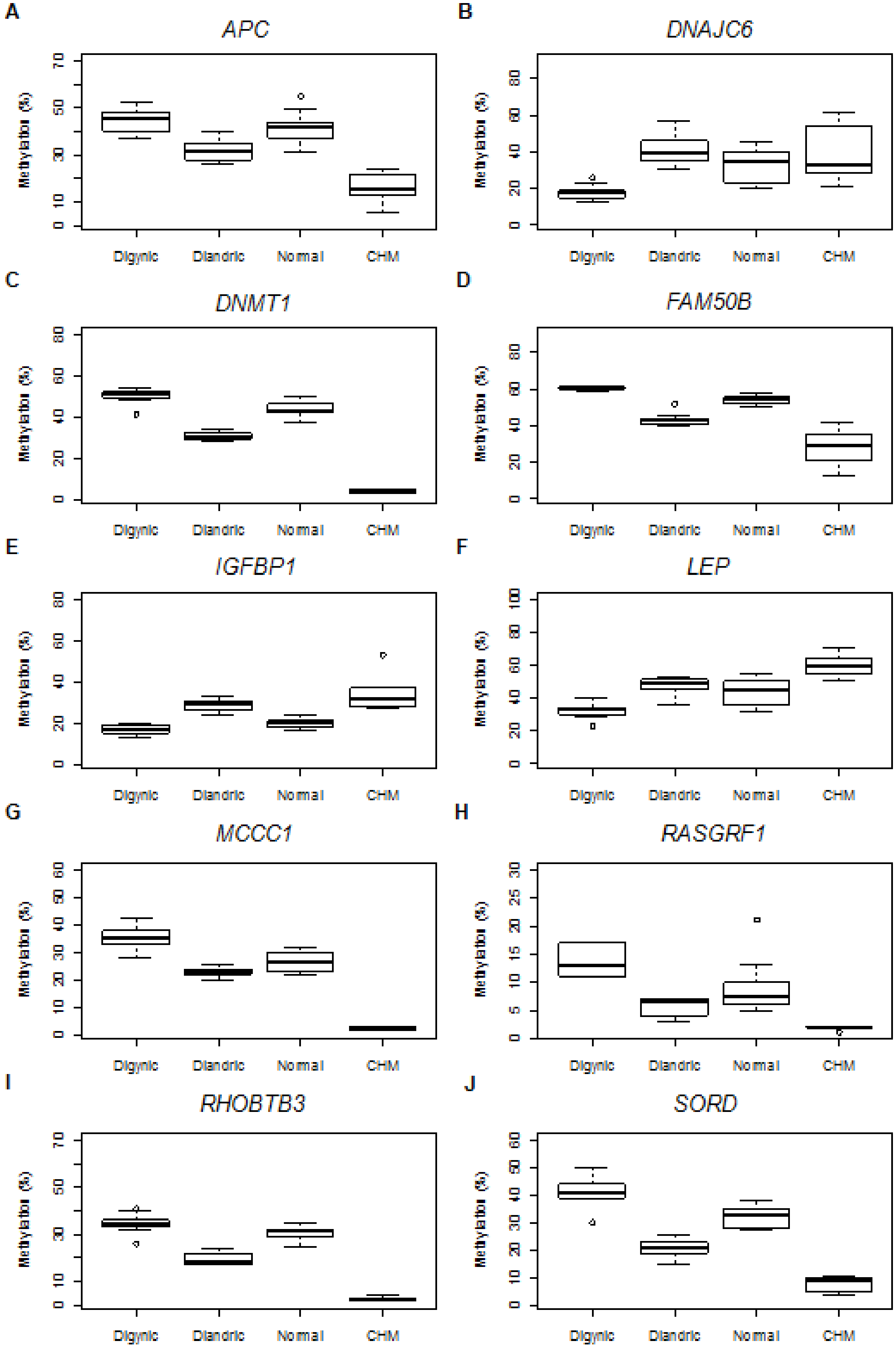
A
APC
Methylation (%)
Digynic
Diandric
Normal
CHM
B
DNAJC6
Methylation (%)
Digynic
Diandric
Normal
CHM
C
DNMT1
Methylation (%)
Digynic
Diandric
Normal
CHM
D
FAM50B
Methylation (%)
Digynic
Diandric
Normal
CHM
E
IGFBP1
Methylation (%)
Digynic
Diandric
Normal
CHM
F
LEP
Methylation (%)
Digynic
Diandric
Normal
CHM
G
MCCC1
Methylation (%)
Digynic
Diandric
Normal
CHM
H
RASGRF1
Methylation (%)
Digynic
Diandric
Normal
CHM
I
RHOBTB3
Methylation (%)
Digynic
Diandric
Normal
CHM
J
SORD
Methylation (%)
Digynic
Diandric
Normal
CHM

Figure S5

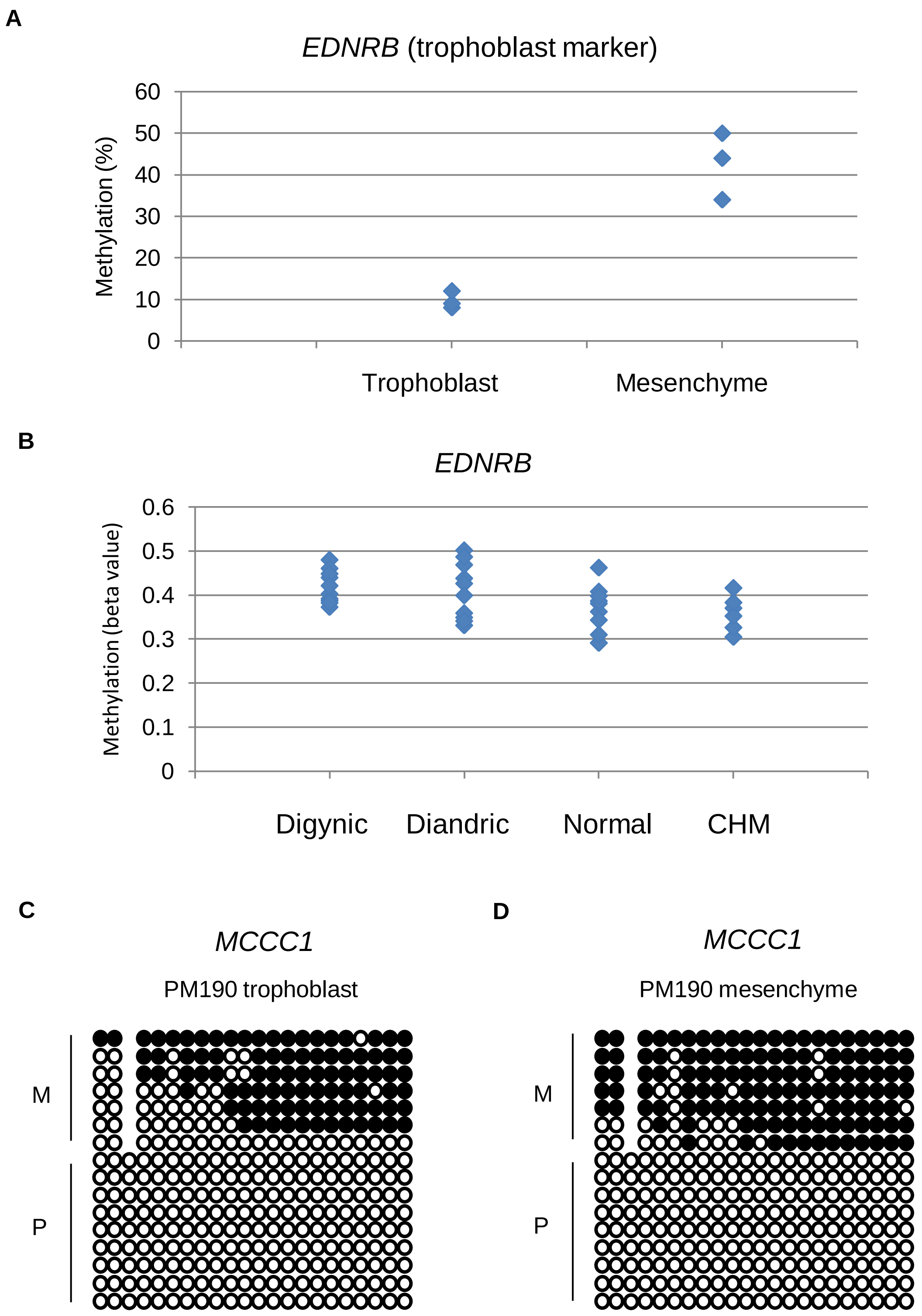
A
EDNRB (trophoblast marker)
Methylation (%)
60
50
40
30
20
10
0
Trophoblast
Mesenchyme
B
EDNRB
Methylation (beta value)
0.6
0.5
0.4
0.3
0.2
0.1
0
Digynic
Diandric
Normal
CHM
C
MCCC1
PM190 trophoblast
M
P
D
MCCC1
PM190 mesenchyme
M
P

Figure S6

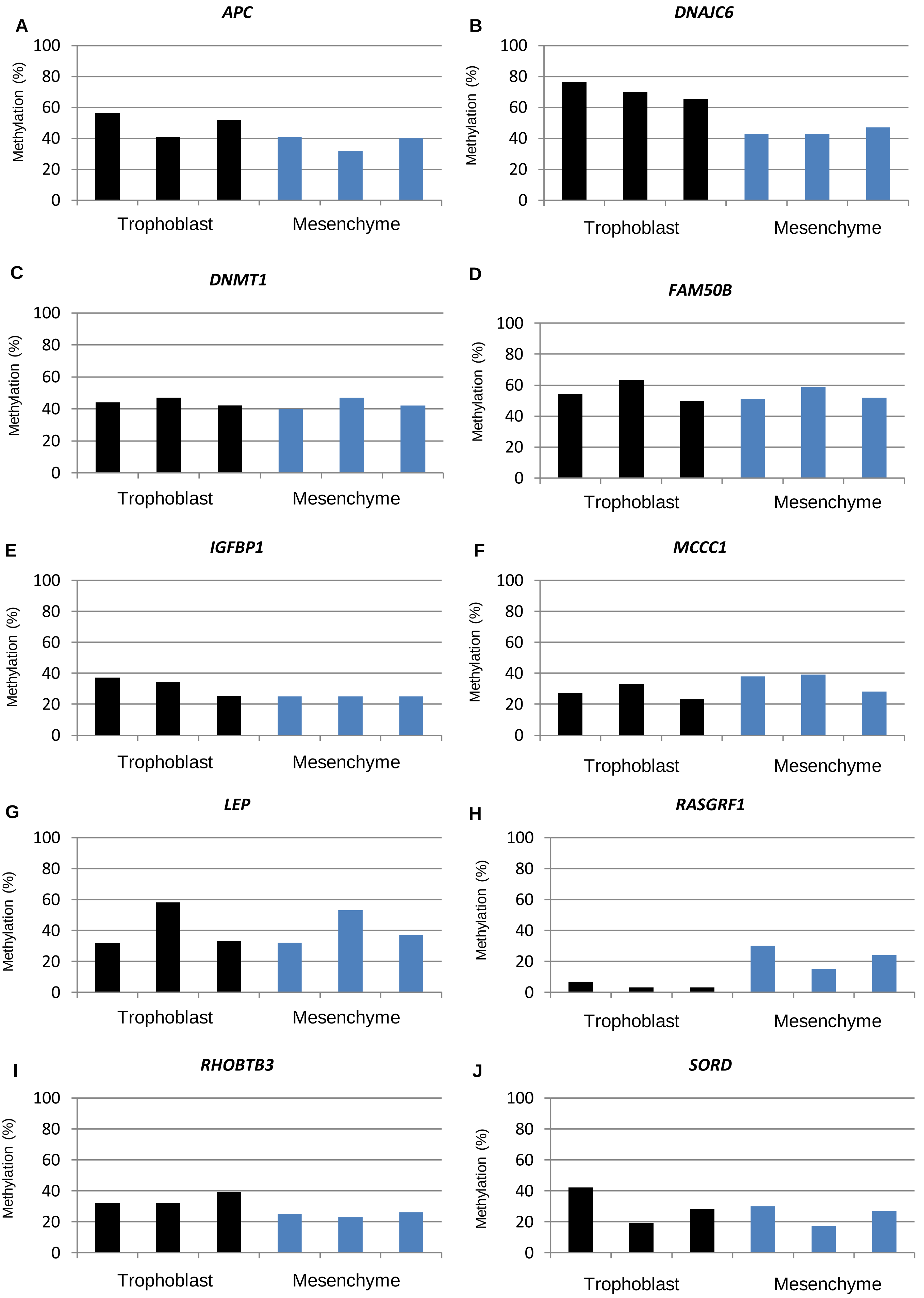
A
APC
B
DNAJC6
C
DNMT1
D
FAM50B
E
IGFBP1
F
MCCC1
G
LEP
H
RASGRF1
I
RHOBTB3
J
SORD
Methylation (%)
0
20
40
60
80
100
Trophoblast
Mesenchyme

Figure S7

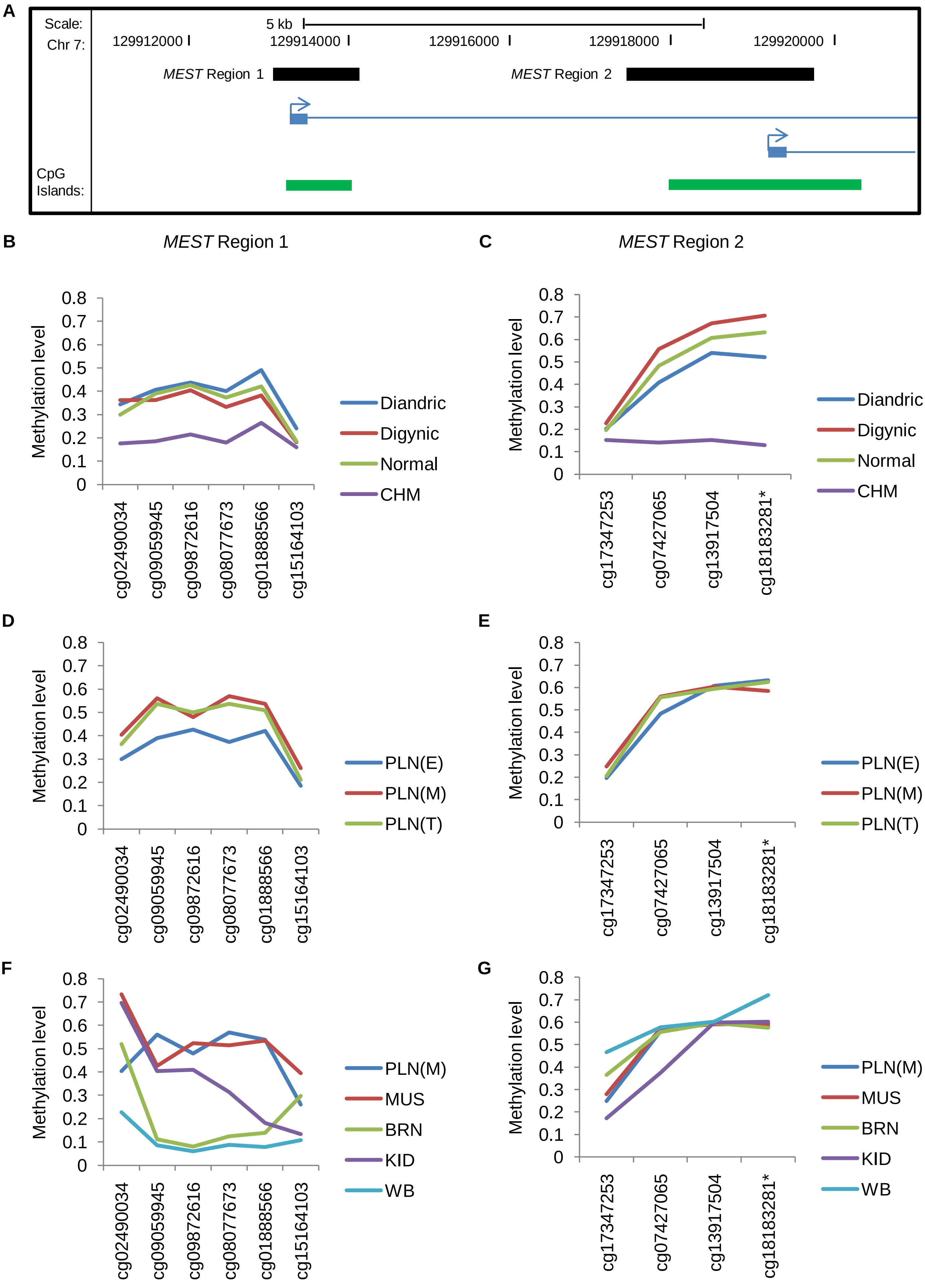
A
Scale:
5 kb
Chr 7:
129912000
129914000
129916000
129918000
129920000
MEST Region 1
MEST Region 2
CpG Islands:
B
MEST Region 1
C
MEST Region 2
Methylation level
0.8
0.7
0.6
0.5
0.4
0.3
0.2
0.1
0
cg02490034
cg09059945
cg09872616
cg08077673
cg01888566
cg15164103
cg17347253
cg07427065
cg13917504
cg18183281*
Diandric
Digynic
Normal
CHM
D
E
PLN(E)
PLN(M)
PLN(T)
F
G
MUS
BRN
KID
WB
